# Comparative performance of precommercial cellulases hydrolyzing pretreated corn stover

**DOI:** 10.1186/1754-6834-4-29

**Published:** 2011-09-07

**Authors:** James D McMillan, Edward W Jennings, Ali Mohagheghi, Mildred Zuccarello

**Affiliations:** 1 National Bioenergy Center, National Renewable Energy Laboratory, Golden, CO, USA

## Abstract

**Background:**

Cellulases and related hydrolytic enzymes represent a key cost factor for biochemical conversion of cellulosic biomass feedstocks to sugars for biofuels and chemicals production. The US Department of Energy (DOE) is cost sharing projects to decrease the cost of enzymes for biomass saccharification. The performance of benchmark cellulase preparations produced by Danisco, DSM, Novozymes and Verenium to convert pretreated corn stover (PCS) cellulose to glucose was evaluated under common experimental conditions and is reported here in a non-attributed manner.

**Results:**

Two hydrolysis modes were examined, enzymatic hydrolysis (EH) of PCS whole slurry or washed PCS solids at pH 5 and 50°C, and simultaneous saccharification and fermentation (SSF) of washed PCS solids at pH 5 and 38°C. Enzymes were dosed on a total protein mass basis, with protein quantified using both the bicinchoninic acid (BCA) assay and the Bradford assay. Substantial differences were observed in absolute cellulose to glucose conversion performance levels under the conditions tested. Higher cellulose conversion yields were obtained using washed solids compared to whole slurry, and estimated enzyme protein dosages required to achieve a particular cellulose conversion to glucose yield were extremely dependent on the protein assay used. All four enzyme systems achieved glucose yields of 90% of theoretical or higher in SSF mode. Glucose yields were reduced in EH mode, with all enzymes achieving glucose yields of at least 85% of theoretical on washed PCS solids and 75% in PCS whole slurry. One of the enzyme systems ('enzyme B') exhibited the best overall performance. However in attaining high conversion yields at lower total enzyme protein loadings, the relative and rank ordered performance of the enzyme systems varied significantly depending upon which hydrolysis mode and protein assay were used as the basis for comparison.

**Conclusions:**

This study provides extensive information about the performance of four precommercial cellulase preparations. Though test conditions were not necessarily optimal for some of the enzymes, all were able to effectively saccharify PCS cellulose. Large differences in the estimated enzyme dosage requirements depending on the assay used to measure protein concentration highlight the need for better consensus methods to quantify enzyme protein.

## Background

The major front-end conversion steps in biochemical platform-based production of cellulosic biofuels are pretreatment and enzymatic hydrolysis [1-3]. Together, these steps work to breakdown the cellulose and hemicellulose found in the cell walls of plants (biomass) to simple sugars ('saccharification'). These sugars then can be converted into biofuel, nominally by fermentation to ethanol, although a variety of other fuel molecules can also be produced from such sugars.

In one of the most widely investigated process options for pretreatment and enzymatic hydrolysis of cellulosic biomass, pretreatment with dilute sulfuric acid is used to solubilize hemicellulosic sugars and increase the reactivity of the remaining insoluble lignocellulosic solids to enzymatic digestion, and cellulase enzymes are then used to hydrolyze the cellulose to fermentable glucose [4-6]. In other process options under development, such as those based on alkaline pretreatments which do not solubilize hemicellulose, the enzymatic hydrolysis step also targets hemicellulose and a broader suite of enzymes is required to hydrolyze both cellulose and hemicellulose components to fermentable sugars [7,8]. The process configuration for enzymatic hydrolysis can be standalone (that is, in the context of an overall process based on sequential hydrolysis and fermentation (SHF)) or it can be combined with fermentation (that is, in the context of an overall process based on simultaneous saccharification and cofermentation (SSCF)), or it can be some hybrid variation falling inbetween SHF and SSCF [9,10].

The cost of enzymes for saccharifying lignocellulosic biomass has been dramatically decreased over the past decade, with US Department of Energy (DOE) cost-shared subcontracts to Genencor and Novozymes spanning 1999 to 2005 decreasing projected enzyme cost approximately 20-fold [11-13]. Despite this substantial accomplishment, biomass saccharification remains a key cost barrier [14] and further reduction in enzyme cost is needed to achieve the DOE Biomass Program's goal to develop and demonstrate cost-competitive cellulosic ethanol production technologies by 2012 [15-17]. Recent state of technology (SOT) cost estimates indicate that enzymes remain the second largest contributor to operating cost in the process, after feedstock, representing an estimated cost of approximately US$0.30 to US$0.50 per gallon of ethanol [6,18].

Recognizing the need to achieve further enzyme cost reduction, the DOE issued Funding Opportunity Announcement (FOA) DE-PS36-07GO97034, 'Development of Saccharifying Enzymes for Commercial Use'. Through a competitive selection process multiyear financial assistance awards were ultimately granted to Danisco, DSM, Novozymes and Verenium [19]. These awards total over US$30 million in DOE cost-shared investment and seek to enable, by 2012, an enzyme cost of approximately US$0.12/gallon ethanol based on a 90% enzymatic hydrolysis sugar yield (from cellulose and hemicellulose).

The National Renewable Energy Laboratory (NREL) is supporting DOE by independently validating enzyme improvements achieved by awardees under this FOA. Part of the validation function is to evaluate and report on an 'anonymous' basis the performance of the four companies' benchmark enzyme systems under identical conditions on a common pretreated feedstock, NREL-prepared dilute acid pretreated corn stover (PCS). These precommercial enzyme systems represent the starting points for the companies' respective, ongoing enzyme cost reduction efforts under this FOA and may differ from final fully formulated commercial products. The FOA requires the performance of each company's benchmark enzyme preparation to be assessed under common conditions and results publicly reported in a non-attributed manner [20]. We satisfy this requirement here by reporting on the performance of the four benchmark cellulase preparations in both SSF and standalone enzymatic hydrolysis modes. These enzyme preparations were obtained under material transfer agreements in which NREL was only permitted to perform performance testing on the specified PCS substrate and measure protein concentrations and key enzyme activities. We were not able to test these preparations on other pretreated substrates nor analyze their component enzyme make-ups or chemical formulations.

## Results

The objective of this study was to characterize the performance of four benchmark cellulose preparations under common hydrolysis reaction conditions using a standard pretreated biomass substrate: NREL dilute acid pretreated corn stover (PCS). The PCS whole slurry contained 32.7% w/w total solids, which corresponds to 17.1% w/w insoluble solids. Table 1 summarizes the composition of the PCS solids and hydrolysate liquid that make up the PCS whole slurry. On a dry basis, the PCS solids contain approximately 59% w/w cellulose (glucan) and 30% w/w lignin.

**Table 1:** Composition of pretreated corn stover solids and liquid fractions

Fraction/content	Components								
**Solid fraction**	**Ash**	**Protein**	**Lignin**	**Glucan**	**Xylan**	**Galactan**	**Arabinan**	**Fructan**	

Concentration (% dry weight)	3.6	0	29.8	58.9	3.4	0.5	0.7	0	

**Liquid fraction**	**Cellobiose**	**Glucose**	**Xylose**	**Fructose**	**Arabinose**	**Galactose**	**Acetic acid**	**HMF**	**Furfural**

Concentration (g/l)	2.6	26.8	81.2	4.5	11.7	6.3	16.7	3.4	2.4

Total sugars (g/l)		29.3	89.9		13.4	6.4			

Oligomeric sugars (g/l)		2.5	8.7		1.7	0.5			

HMF = hydroxymethylfurfural.

Shake flask scale experiments were conducted to develop dosage response curves for each enzyme acting in both SSF and enzymatic hydrolysis only (EH) reaction modes. Data were generated to describe how the extent of cellulose conversion to glucose for each enzyme preparation (that is, monomeric glucose yields as a percent of theoretical maximum glucose recovery based on input cellulose) varied as a function of the amount of enzyme protein loaded, with total enzyme protein measured using either bicinchoninic acid (BCA) or Bradford protein assay.

### Enzyme preparation protein concentration

**Figure 1:**
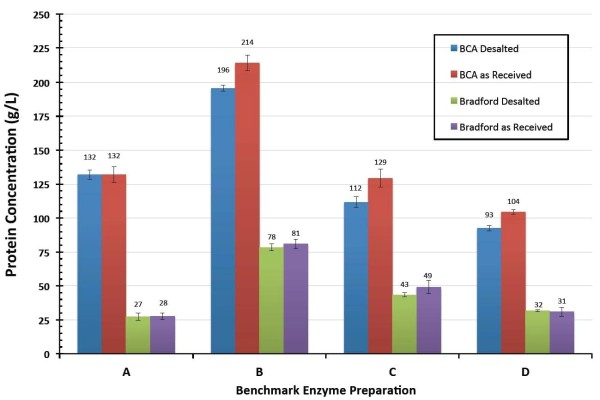
**Protein concentrations measured by bicinchoninic acid (BCA) and Bradford assays for each preparation**.

All enzyme preparations were assayed for their total protein content using both BCA and Bradford protein assays calibrated against bovine serum albumin (BSA). Results obtained on both as received and desalted enzyme samples are summarized in Figure 1. For all enzyme preparations, only comparatively small differences were observed in apparent protein concentrations of enzyme samples as received or after being processed ('desalted') to remove low molecular weight components that might otherwise cause interferences. For the desalted samples, the apparent protein concentration measured by Bradford assay varied from a low of approximately 30 g/l in preparations A and D to a high of almost 80 g/l in preparation B. In contrast, apparent protein concentrations measured by BCA assay on desalted samples ranged from a low of 93 g/l in preparation D to a high of 196 g/l in preparation B.

Our reference method is to apply the BCA assay to desalted enzyme samples, and for the remainder of this paper we will only refer to protein concentration measurements based on desalted enzyme samples analyzed by BCA or Bradford assay unless otherwise stated.

### Enzyme preparation hydrolytic activity

The cellulase activity of each preparation (performed on desalted enzyme samples) was also evaluated for β-glucosidase activity and filter paper activity, as summarized in Table 2. The lowest β-glucosidase activity level, 69 IU/ml, was observed in preparation D and the highest, 741 IU/ml, in preparation B. Levels of filter paper activity, a measure of the cellulose hydrolyzing potential of an enzyme preparation, varied from a low of 18 filter paper activity units (IFPU)/ml in preparation A to a high of 105 IFPU/ml in preparation B.

**Table 2:** β-Glucosidase and filter paper activities

Enzyme	β-Glucosidase activity, IU/ml	Filter paper activity, IFPU/ml
A	233	18

B	741	105

C	259	46

D	69	41

IFPU = Filter paper activity units.

### Enzyme performance: enzymatic hydrolysis in SSF of washed PCS

In SSF hydrolysis mode, no free glucose (or other free sugar) is initially present because the substrate is washed PCS solids. No glucose accumulates during SSF because as glucose is produced, by enzymatic hydrolysis of cellulose, it is fermented to ethanol (and carbon dioxide). Thus, in the SSF system the extent of cellulose conversion to glucose is back calculated from the amount of ethanol produced.

**Figure 2:**
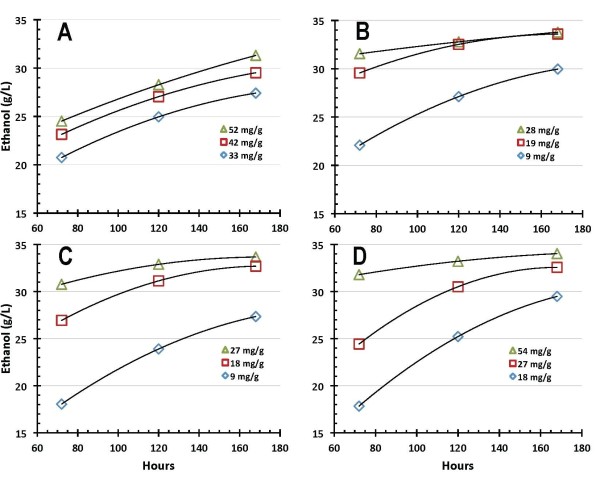
**Ethanol production during simultaneous saccharification and fermentation (SSF) mode enzymatic hydrolysis for enzyme preparations A, B, C and D**. Enzyme loadings are listed in the figure legends and are based on protein concentrations measured using the bicinchoninic acid (BCA) assay.

**Figure 3:**
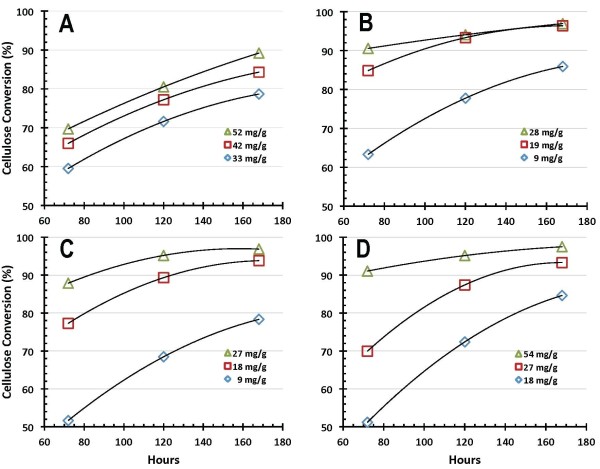
**Cellulose conversion to glucose during simultaneous saccharification and fermentation (SSF) mode enzymatic hydrolysis for enzyme preparations A, B, C and D, respectively**. Enzyme loadings are listed in the figure legends and are based on protein concentrations measured using the bicinchoninic acid (BCA) assay.

Representative profiles for ethanol production and cellulose conversion obtained in SSF shake flask experiments performed at pH 5 and a temperature of 38°C using the four enzyme preparations are shown in Figures 2 and 3, respectively. Note that these plots only show a subset of acquired SSF performance data in order to convey general trends. Also, the data trend lines shown in this and subsequent figures are for illustration only. Enzyme A performance in SSF, ethanol production shown in Figure 2A and cellulose conversion in Figure 3A, is depicted at (BCA assay-based) protein loadings of 33, 42 and 52 milligrams of enzyme protein per gram cellulose initially loaded (mg/g hereafter). The SSF performance of enzyme B is correspondingly depicted at enzyme doses of 9, 19 and 28 mg/g, with ethanol production shown in Figure 2B and cellulose conversion in Figure 3B. Similarly, the performance of enzyme C is shown at enzyme loadings of 9, 18 and 27 mg/g (ethanol production in Figure 2C and cellulose conversion in Figure 3C), and that for enzyme D is shown at a loading of 18, 27 and 54 mg/g (ethanol production in Figure 2D and cellulose conversion in Figure 3D). As Figure 2 illustrates, levels of ethanol production increase with time from 3 days (72 h) to 7 days (168 h), and with enzyme dosage. As Figure 3 shows, the benefits of increased enzyme loading diminish as higher extents of conversion are reached. This is most clearly seen here in enzyme preparations B and C, where increasing the enzyme dosage from approximately 18 mg/g to approximately 28 mg/g results in considerably less benefit to ethanol production (Figures 2B and 2C) or cellulose conversion (Figures 3B and 3C) than is observed when the dosage increases from approximately 9 mg/g to approximately 19 mg/g. Despite differences in absolute enzyme loading levels and maximum extents of ethanol production and cellulose conversion reached, Figures 2 and 3 show that all four enzyme systems perform well in SSF mode and have the capability to achieve cellulose to glucose conversion levels of 90% or higher in 7 days or less provided they are dosed at sufficiently high levels.

**Figure 4:**
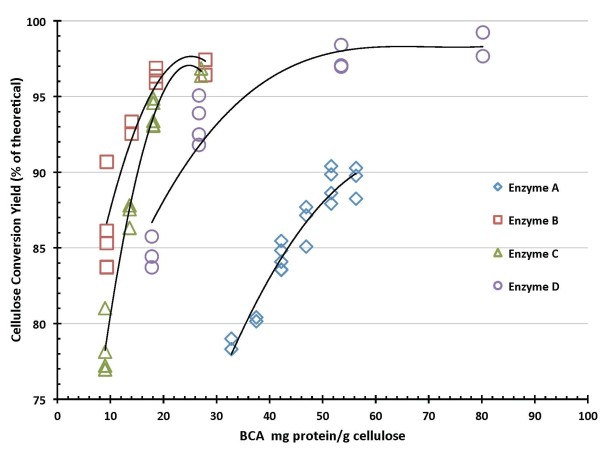
**Cellulose conversion to glucose in simultaneous saccharification and fermentation (SSF) hydrolysis mode as a function of enzyme protein loading measured by bicinchoninic acid (BCA) assay**.

**Figure 5:**
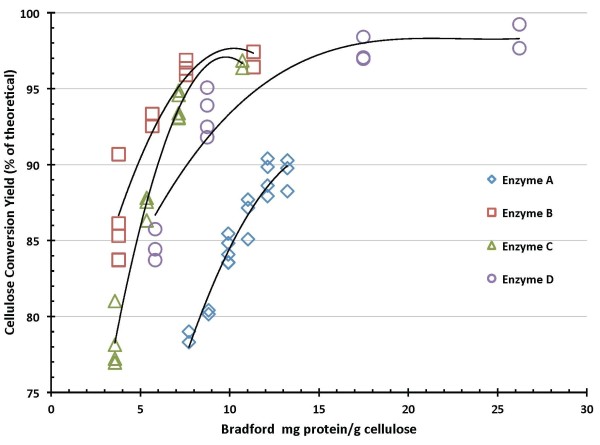
**Cellulose conversion to glucose in simultaneous saccharification and fermentation (SSF) hydrolysis mode as a function of enzyme protein loading measured by Bradford assay**.

Figures 4 and 5 summarize 7-day SSF cellulose conversion results as a function of enzyme loading. Figure 4 shows these results for enzyme protein loadings based on BCA protein assay measurements, and Figure 5 shows the same results based on Bradford assay protein measurements. Regardless of which protein assay is used, enzyme preparations B and C exhibit the best performance in SSF under the conditions tested (pH 5, 38°C), achieving cellulose conversion levels to glucose above 90% of theoretical at the lowest enzyme dosages (roughly 10-15 mg/g based on BCA, 4-6 mg/g based on Bradford). Enzyme D shows a lower dosage response than enzymes B and C but achieves cellulose conversion levels above 90% at higher enzyme loadings. Enzyme A is the poorest performer under these SSF hydrolysis conditions, requiring higher loadings than the other preparations to reach cellulose conversion levels above 80%. Moreover, in contrast to the other enzyme systems, in SSF mode enzyme A was not able to hydrolyze cellulose to conversion levels much above 90%; all of the other systems showed the ability to reach cellulose conversion levels above 95% of theoretical at sufficiently high enzyme loadings.

### Enzyme performance: enzymatic hydrolysis in PCS whole slurry

In standalone enzymatic hydrolysis (EH) mode, free sugars produced in the dilute acid pretreatment step are initially present (except in the case where washed PCS solids are used as the substrate rather than whole slurry (WS)). In the EH reaction mode, glucose and possibly higher gluco-oligomers (for example, cellobiose, cellotriose, and so on) accumulate and potentially cause product feedback inhibition of the reaction as hydrolysis proceeds. The extent of cellulose conversion to glucose is calculated from the amount of (net) monomeric glucose produced.

**Figure 6:**
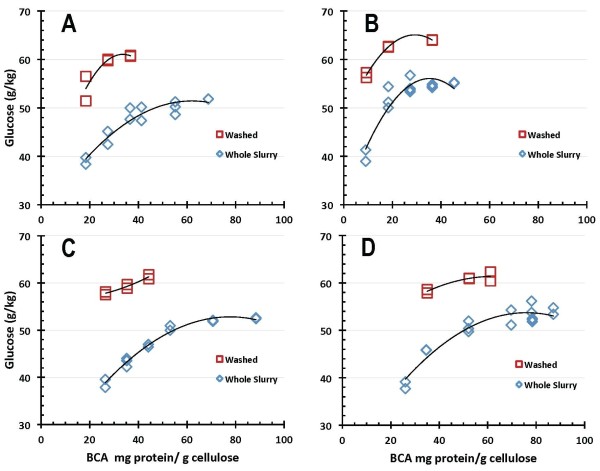
**Glucose production in enzymatic hydrolysis mode on washed pretreated corn stover (PCS) solids and unwashed whole slurry PCS as a function of protein loading measured by bicinchoninic acid (BCA) assay**.

**Figure 7:**
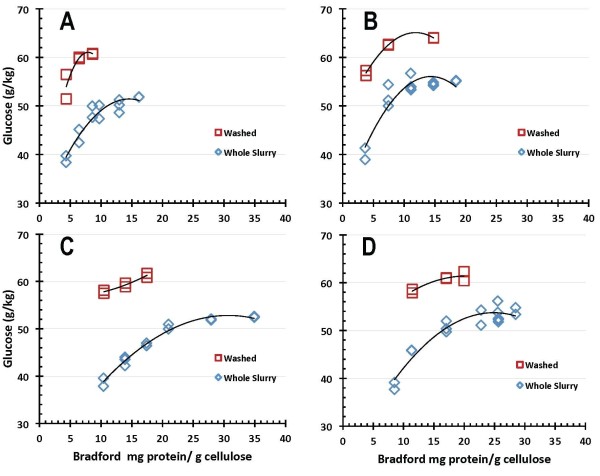
**Glucose production in enzymatic hydrolysis mode on washed pretreated corn stover (PCS) solids and unwashed whole slurry PCS as a function of protein loading measured by Bradford assay**.

Comparative 7-day EH glucose production results obtained on both washed and unwashed PCS for the four enzyme systems are shown as a function of enzyme dosage level in Figures 6 and 7, with enzyme loadings reported on a BCA protein assay basis in Figure 6 and on a Bradford protein assay basis in Figure 7. More differentiation between the four enzyme systems was seen in the EH reaction mode under the tested conditions (pH 5, 50°C), albeit all enzyme systems performed substantially better on washed PCS solids than on PCS WS. Glucose production levels above 60 g/kg were achieved by all four enzyme preparations hydrolyzing washed PCS solids, whereas in PCS WS the highest levels of (net) glucose production are lower, reaching only 50-55 g/kg, and larger doses of enzymes are needed to achieve this. At equivalent total enzyme protein loadings, glucose production levels are approximately 10-15 g/kg higher on washed PCS solids than on PCS WS. Enzyme B exhibits the best performance in this hydrolysis mode, reaching the highest levels of glucose production and showing saturation behavior at lower enzyme loadings than enzymes A, C or D.

**Figure 8:**
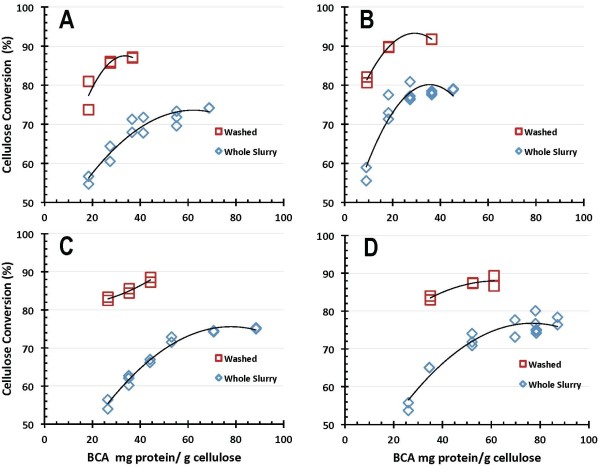
**Cellulose conversion to glucose as a function of enzyme protein loading measured by bicinchoninic acid (BCA) assay**.

**Figure 9:**
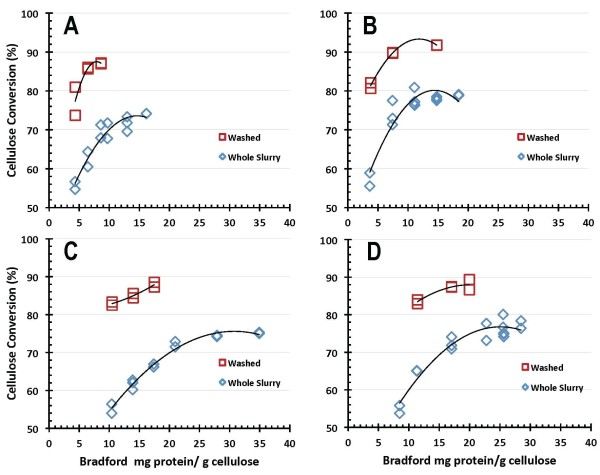
**Cellulose conversion to glucose as a function of enzyme protein loading measured by Bradford assay**.

The process yield implications of these results are more clearly shown in Figures 8 and 9, which plot the corresponding 7-day cellulose conversion yields for the four benchmark enzyme systems, with enzyme loadings reported on a BCA protein assay basis in Figure 8 and on a Bradford protein assay basis in Figure 9. The trends are similar to those discussed above, with enzyme B exhibiting somewhat better performance than enzymes A, C and D. Again, cellulose conversion performance is substantially better on washed PCS solids than on the PCS WS, with all of the enzyme systems achieving maximum yields of cellulose conversion to glucose above 85% to 90% of theoretical hydrolyzing washed PCS solids but only reaching maximum yields of 75% to 80% hydrolyzing PCS WS.

Selected enzymatic hydrolysis results were subsequently replicated at the 500 g scale. While these experiments confirmed the four enzymes' relative performance attributes for cellulose conversion and glucose production that had been observed at similar enzyme loadings at the 100 g scale, absolute performance levels obtained at the 500 g scale were modestly different than what had been observed at the smaller scale. Three of the enzymes (B, C and D) achieved roughly 5% higher cellulose conversion to glucose at the larger scale compared to the 100 g scale, and one enzyme (A) achieved about 5% lower cellulose conversion to glucose performance (data not shown).

## Discussion

It needs to be emphasized that the obtained results are for the benchmark enzyme preparations that represent the starting points for Danisco's, DSM's, Novozymes' and Verenium's respective enzyme cost reduction and activity improvement projects. These projects are expected to have made significant progress since initially awarded, meaning the level of cellulase performance presently available is likely to be considerably better than reported here for the benchmark cellulase preparations. Moreover, the results reported here show comparative performance under specific SSF and EH conditions that are likely not optimal for some of the enzymes tested. Nonetheless, the results presented here using a common set of SSF and EH test conditions provide a baseline against which future advances can be compared.

Experiments were designed to assess enzyme performance in both SSF and EH modes, since both of these configurations for enzymatic hydrolysis are leading biorefining process options under active development. Both SSF and SHF (EH mode) offer potential advantages and the decision as to which approach is preferable for a particular situation is complex, depending on multiple factors including the cost and performance attributes of the enzymes as well as the nature of the integrated process in which they will be applied.

SSF-based processing of washed pretreated solids minimizes the initial amount of background inhibitors present and also the accumulation of sugars during the hydrolysis reaction, thus potentially enabling higher cellulose conversion yields to be obtained than are possible in EH. This is evident from the comparative performance results reported here. In SSF hydrolysis mode using washed PCS solids, all four of the enzyme systems achieved cellulose conversion to glucose yields of 90% of theoretical or higher, with enzymes B and C showing the best performance attributes regardless of which protein assay method was used (Figures 4 and 5). By maintaining low sugar concentrations, SSF also has the potential benefit of reducing the likelihood or extent of contaminating microorganisms. However, SSF commits the sugars to be converted into a particular fermentation product (for example, ethanol), whereas an EH (SHF) approach produces sugars as an intermediate product that can be used for its highest value(s). By not committing all of the produced sugars to a particular fermentation product, SHF processing facilitates the ability to produce multiple sugar-based products. SHF also can be advantageous when there are large differences in pH or temperature optima or other operational preferences between the enzyme system and fermentation strain since the saccharification and fermentation reactions can be carried out at their respective optimum conditions. An SHF process also conceivably permits insoluble solids to be removed prior to fermentation, thus potentially enabling biochemical engineering strategies such as cell recycling to be applied to increase fermentor productivity. However, while WS processing by SHF is conceptually appealing, it is a far more challenging system for enzymes to perform well in. This is evident in the results presented here, where performance levels decline in enzymatic saccharification mode compared to SSF mode, with cellulose conversion levels decreasing modestly for EH of PCS washed solids and more significantly for EH of PCS WS. The strong detrimental influence exerted by background components in hydrolysate liquids on enzyme performance is illustrated by cellulose conversion yields (to glucose) at similar enzyme loadings being approximately 10% to 15% lower on PCS WS than on washed PCS solids (Figures 8 and 9).

**Figure 10:**
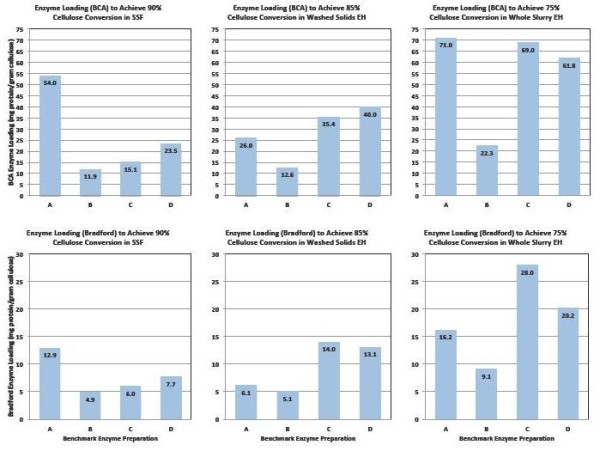
**Estimated enzyme loadings required to achieve conversion levels of 90% in simultaneous saccharification and fermentation (SSF) mode (left panels), 85% in enzymatic hydrolysis of washed pretreated corn stover (PCS) solids (middle panels) and 75% enzymatic hydrolysis of PCS whole slurry (right panels)**. Upper set shows results with protein quantified by bicinchoninic acid (BCA) assay, lower set with protein measured by Bradford assay.

A comparative analysis was carried out to evaluate the approximate enzyme loadings required to achieve different levels of performance across the three enzymatic saccharification process configurations examined in this work. Using results based on both protein assays, estimates were made of the enzyme loadings required to achieve 90% cellulose conversion in SSF mode, 85% in washed solids EH mode and 75% in whole slurry EH mode. This was performed graphically by fitting curves to describe cellulose conversion as a function of enzyme protein loading for each of the test cases and then determining by visual approximation where the curves crossed these targeted conversion levels (analysis not shown). The results of this qualitative comparison are shown in Figure 10, which shows in bar graph form the approximate dosages of each enzyme preparation required to achieve 90% cellulose conversion in SSF, 85% in washed solids EH and 75% in whole slurry EH. This figure shows how differently the four enzyme systems behave across the range of test conditions. It also readily shows how estimated target dosage levels (mg enzyme protein per g cellulose) vary depending on which assay is used to measure the protein concentrations in the enzyme preparations. As Figure 10 illustrates, enzyme B requires the lowest protein loading to achieve the target conversion levels in all cases, but its comparative performance advantage over the other enzyme systems varies substantially across the three test cases as well as depending upon which protein assay is used. In the SSF hydrolyzing washed PCS solids (target cellulose to glucose conversion level of 90%), the four enzymes show similar relative behavior regardless of whether the BCA or Bradford assay is used (Figure 10, upper and lower left panels, respectively), with the order of effectiveness of the four enzymes (under the SSF test conditions) varying highest to lowest B > C > D >> A. In contrast, for enzymatic hydrolysis of washed PCS solids (target conversion level of 85%), the order of efficacy of the four enzyme preparations is B >> A > C > D on a BCA assay basis but changes to B > A >> D > C when protein loading is based on the Bradford assay (Figure 10, upper and lower panel middle panels, respectively). In the enzymatic hydrolysis of PCS WS, the order of effectiveness is B >> D > C > A based on the BCA assay, and changes to B >> A > D >> C based on the Bradford assay (Figure 10, upper and lower right panels, respectively).

There are only limited correlations between the overall performance of the enzyme preparations (Figure 10) and their enzyme activity levels (Table 2). Foremost, the absolute filter paper and β-glucosidase activity levels are highest in enzyme preparation B (Table 2), which is the preparation that performed the best across all of the conditions tested (Figure 10). Beyond this, the predicted order of effectiveness (highest to lowest) but not in relative magnitude is similar to the order observed under SSF test conditions (Figure 10, upper and lower left panels). In contrast, the predicted order of performance based on β-glucosidase activity level alone is B >> C > A >> D (Table 2), a pattern that isn't observed under any of the hydrolysis modes tested. Similarly, the performance order based on the ratio of β-glucosidase to filter paper activity (or its inverse) would be A >> B > C >> D (or the reverse), which also isn't seen under any of the conditions tested. These results show the limitations of trying to use activity measurements such as the filter paper assay to predict the differential performance of enzyme preparations in actual hydrolysis processes.

As Figure 10 illustrates, there is great diversity in the relative performance of the benchmark enzymes depending upon which hydrolysis mode and protein assay basis are examined. The wide variation in relative efficacy at least partially reflects that each of the enzymes are being developed to perform optimally on a specific pretreated feedstock in a particular hydrolysis mode at defined reaction conditions that in some cases are different than tested here. The overall process the enzyme is being used within also may be targeting conversion levels different than evaluated here. Overall, the wide variation in absolute performance levels suggests that there are large differences among the four benchmark preparations in their respective sensitivities to other factors influencing enzymatic hydrolysis rates and yields, for example, the levels of inorganic salts and organics such as acetic acid, furfural and soluble lignin as well as insoluble lignin, which together with other hydrolysate components modulate water activity and enzyme adsorption and desorption phenomena.

### Protein quantitation

Reliable measurement of protein concentration is useful to be able to accurately dose enzyme and estimate enzyme cost for different enzyme preparations. For enzyme-based biorefining to advance, it is essential to be able to carry out meaningful comparative assessments of the performance and specific activity attributes of different enzyme preparations under a range of operational conditions. However, protein measurement is complicated, with results influenced by the type of proteins being quantified as well as the nature of the solution matrix in which they are suspended and the type of protein against which they are calibrated [21-24]. An important issue identified in the course of this work is that efficient, cost effective consensus methods for measuring protein concentration still do not exist [25]. Spectrophotometric dye-based assays such as the BCA and Bradford methods employed in this study are useful in being reproducible and widely available, but they are confounding because the results they provide are inconsistent with one another. This is a consequence of these assays' different chemistries and sensitivities to interfering compounds [21-24]. Moreover, these assays measure total protein content not just cellulase enzyme protein content, and this further complicates the interpretation of the results reported here.

In this work, we measured significant differences in protein concentrations between the different enzyme samples as well as large differences in apparent concentrations depending on which assay was used. As Figure 1 illustrates, for the four enzyme preparations evaluated, the BCA assay consistently measured apparent protein concentrations roughly 2.6 to 4.8 times higher than the Bradford assay. The consequence is correspondingly large differences in estimated absolute enzyme dosage levels depending upon which assay is used to measure the concentration of the enzyme preparations. These findings highlight the need to develop an accepted standard method for directly determining protein concentration that is easier to apply than the relatively more time consuming and expensive approach of quantitative amino acid analysis. Our results vividly show that wide differences in results are possible using standard direct assay methods and highlight how misleading it can be to compare results obtained using different assays, particularly across a range of industrial enzyme samples expected to exhibit wide differences in composition [26]. Beyond this, the obtained results may also reflect limitations in using BSA as a protein calibration standard for cellulases.

### Conversion performance data quality

There is appreciable scatter in the 7-day cellulose conversion results for both hydrolysis modes investigated, SSF (Figures 4 and 5) and EH (Figures 8 and 9). However, control flasks run with each experimental block demonstrated good reproducibility, with the calculated coefficient of variance below 3% (data not shown). This variability likely reflects difficulties in representatively sampling experimental flasks containing high levels of insoluble solids, coupled with inaccuracies in analytical techniques. How to best measure cellulose conversion or glucose production yields in such systems remains an open question and an active area of investigation [27-29].

The reasons for the reproducible discrepancies in absolute performance levels between the 100 g and 500 g experiments are unclear and require further study. Two hypotheses are that differences in reaction hydrodynamic environment or reaction temperature (or both) are playing a role, and other as yet unrecognized factors might also be contributing. The fact that no sampling was performed during the 500 g experiments (only at the beginning and end) means that these reactions remained at their target temperature for the duration of an experiment. In contrast, the 100 g experiments were sampled at 3 and 5 (and 7) days and thus likely experienced minor reductions in reaction temperature during sampling, since this occurred at ambient temperature in a sterile laminar flow hood. There are also differences in the mixing hydrodynamics between the 250 ml (100 g) and 1 l (500 g) reaction bottles.

## Conclusions

This study contributes to the literature by providing an extensive amount of quantitative information about the performance of four industrial benchmark cellulase preparations. Comparative testing results on PCS demonstrate that all four of these enzyme systems function well as complete cellulases capable of achieving high levels of cellulose conversion. Significant performance differences were observed among the four enzyme systems, however. Results show that achievable glucose yields and the enzyme dosages required to reach them vary widely between the enzyme preparations and also depend on hydrolysis mode, with better performance observed on washed PCS than on WS PCS.

In SSF mode, all four enzyme systems achieved glucose yields from cellulose of 90% of theoretical or higher. The enzyme dosages required to achieve this level of conversion ranged from 12 mg enzyme protein/g cellulose to 54 mg/g on a BCA protein assay basis, and from 5 mg/g to 13 mg/g on a Bradford protein assay basis. Glucose yields were reduced in EH mode, with all enzymes achieving glucose yields of at least 85% of theoretical on washed PCS solids and 75% in PCS whole slurry. For EH mode, reaching cellulose conversion to glucose yields of 85% on washed PCS solids required enzyme dosages of 13-40 mg/g on a BCA basis and 5-14 mg/g on a Bradford basis. Achieving glucose yields of 75% on PCS whole slurry required enzyme loadings of 22-71 mg/g on a BCA basis and 9-28 mg/g on a Bradford basis. One of the enzyme preparations ('enzyme B') exhibited the best absolute performance in terms of attaining high conversion yields at lower enzyme loadings under all conditions tested, but the relative performance of the four enzyme systems to one another varied significantly depending upon which hydrolysis mode and protein assay were used as bases for comparison.

Estimates of enzyme protein dosages (mg enzyme protein per g cellulose) required to achieve specific levels of cellulose conversion were highly dependent upon which protein assay (BCA or Bradford) was used to quantify protein concentration, with BCA assay-based estimates roughly threefold to fivefold higher than those based on the Bradford assay. This large difference in apparent protein dosage requirement depending upon which assay is used motivates the need to develop consensus standard methods for quantifying enzyme protein.

The enzyme systems reported on here are being further improved upon through ongoing DOE cost-shared projects aimed at reducing enzyme cost to enable economical production of biochemically-based cellulosic biofuels and chemicals. Moreover, some of these enzyme systems may perform better under operating conditions different than were tested in this study. Thus, the results reported here likely underestimate the level of enzyme performance available in the marketplace today. Nonetheless, these results provide a useful quantitative baseline against which future enzyme improvements can be compared.

## Methods

Comparative performance tests were designed to show in a non-attributed manner how Danisco's, DSM's, Novozymes' and Verenium's benchmark enzyme preparations perform on NREL dilute-acid PCS. Two modes of enzymatic hydrolysis were tested, SSF on washed PCS solids and standalone EH using both PCS WS and washed solids. The EH whole slurry evaluations were performed at a target total solids loading of 20% w/w total solids, which corresponded to an insoluble PCS solids loading of 10.4% w/w and a cellulose loading of 6.2% w/w. The SSF and EH experiments performed on washed solids were carried out at a total (and insoluble) solids loading of 10.4% w/w and a cellulose loading of 6.2% w/w. The experiments generally followed the shake flask method described previously [30] and incorporated appropriate replicates and controls.

### Biomass substrate preparation

The corn stover used in this study was harvested in the fall of 2003 from a farm in northeastern Colorado, USA. To increase its susceptibility to enzymatic hydrolysis, it was treated with dilute sulfuric acid and steam in a 900 dry kg/day pilot-scale continuous reactor operating at an input solids concentration of 30% w/w. This 'pretreatment' reaction was carried out at a temperature of 190°C using an acid concentration of 0.048 g acid/g dry biomass and a residence time of approximately of 1 min. Details about this continuous direct steam injection pilot scale pretreatment system have been described previously [5]. The acidic (approximately pH 1.2) pretreatment slurry contained 32.7% w/w total solids (17.1% w/w insoluble solids) and was stored at 4°C prior to use. The average composition of the insoluble solids and liquid fractions comprising this material, NREL PCS lot P080828CS, is summarized in Table 1.

The pH of the acidic PCS WS was adjusted to pH 5.0 using NH_4_OH prior to the addition of enzymes. Under constant mixing, the slurry's pH was raised from an initial pH of approximately 1.2 to the target value of 5.0 by adding concentrated NH_4_OH (29.8% assayed as NH_3_; JT Baker, Phillipsburg, NJ, USA). A representative batch pH adjustment to produce 2 kg of 20% total solids (factoring in the enzyme dosing) pH 5.0 WS consisted of diluting 1.2 kg of WS at total solids approximately 32% w/w to a final total solids level of 20% (w/w) using a combination of NH_4_OH (approximately 32.0 ml NH_4_OH per 1200 g WS) and sterile deionized water. The pH-adjusted WS was then stored at 4°C overnight to ensure pH equilibration, and thereafter was maintained at 4°C until it was used for experimentation over a subsequent 2-week period. No steps beyond hygienic laboratory technique (for example, autoclaving or addition of antimicrobial compounds) were taken to avoid microbial contamination.

To prepare for experiments using washed solids, whole slurry PCS was washed using a basket centrifuge (Model STM-2000, Western States, Hamilton, OH, USA) with a nylon filter bag (105 μm pore size, 25% open area, Sefar filtration cloth, Western States) until the glucose concentration in the wash water was below 0.1 g/l. The washing procedure consisted of adding 4.30 kg of the whole slurry to 12.25 kg of domestic water. After thoroughly mixing, the diluted WS was pumped to the centrifuge. The centrifuge was operated at 300 rpm (17.9 *g*) until sufficient solids were deposited on the filter bag inside the basket centrifuge. During this time the supernatant exiting the centrifuge was recycled (twice) to capture fines that came through, although some fines were lost during the subsequent washing step. Washing of the filter bag cake layer with domestic water was then performed using a centrifuge speed of 1800 rpm (644.0 *g*). After the washing step was complete, the centrifuge speed was further increased to 3500 rpm (2435.1 *g*) for 30 min to increase the extent of dewatering of the washed substrate. Samples of the supernatant exiting the centrifuge were taken at 30 and 60 min during the wash and upon reaching 3500 rpm to verify that the glucose concentration remained less than 0.06 g/l. After the final dewatering cycle, the washed solids were recovered, mixed well, sampled for insoluble solids analysis, and then placed in a container and stored at 4°C until use. The total solids recovery in the washing step was approximately 1.70 kg (91% recovery based on insoluble solids). The insoluble solids, measured gravimetrically after drying overnight at 105°C, were determined to be 39.1% w/w.

### Enzyme protein and activity assays

Enzyme preparations were assayed both as received and after desalting using a 5 kDa cut off filter to remove potentially interfering low molecular weight components, for example, salts, small peptides and media components that might otherwise compromise enzyme protein and activity measurements [21-24,31]. The desalting procedure was performed on 2 ml undiluted sample aliquots using a HiPrep 26/10 desalting column with a Sephadex G-25 column matrix using 50 mM acetate buffer at pH 5.0 and a flow rate of 10 ml/min. The 2 ml calibrated sample loop was overloaded to ensure it contained only the enzyme preparation to be desalted. Following sample injection, the loop was washed with an additional 1.0 ml of buffer before being taken offline. Proteins within the void volume of the column (approximately 20 ml) were collected and pooled for subsequent protein and activity measurements.

Protein concentration was measured using the BCA and Bradford assays, using BSA as standard protein, to determine dosage levels in SSF and enzymatic hydrolysis experiments [30]. Protocols supplied by the manufacturer (BCA and Bradford Protein Assay Kit, Pierce, IL, USA) were closely followed. The BCA assay, which was used as our reference protein measurement method based on earlier work [25], was performed as follows: A set of BSA protein standards was developed following the dilution scheme described for the 'Standard Test Tube Protocol' (working protein concentration range = 20-1,500 μg/ml). Unknown sample replicates along with the BSA standards were pipetted into labeled test tubes and mixed well. Numerous dilutions were performed on the unknown samples to achieve protein concentrations in the range of approximately 150 μg/ml to 350 μg/ml (OD_562_, approximately 0.2-0.5 absorbance units). Tubes were covered and incubated at 37°C for 30 min, then cooled to room temperature using chilled water, vortexed and absorbance measured within 10 min. The average absorbance of the blank standard replicates read at 562 nm was subtracted from the absorbance measured for all individual standards and unknown sample replicates. A standard curve was prepared by plotting the average blank-corrected 562 nm absorbance of each BSA standard versus its protein concentration in μg/ml. This standard curve was then fitted with a second-order polynomial best-fit regression equation that was then used to determine the protein concentration of each unknown sample. Several measurements were performed of each enzyme sample, in triplicate, to ensure protein concentration results were reproducible. Filter paper activity (IFPU) and β-glucosidase activity were also measured for each preparation. The filter paper assay was performed as described in detail elsewhere [32,33]. β-Glucosidase activity was also measured using a previously established protocol [34].

### Simultaneous enzymatic hydrolysis and fermentation

The efficacy of the enzyme systems for hydrolyzing washed PCS lignocellulosic solids in the presence of ethanol fermentation was tested by shake flask scale SSF as described previously [30]. Briefly, the thoroughly washed PCS solids (described above) were loaded into 125 ml Erlenmeyer flasks to achieve a final concentration of 6.2% (w/w) cellulose (about 10% w/w insoluble solids). Flasks were filled with solids and water, weighed, autoclaved for 30 min, cooled and reweighed to establish the amount of water evaporated during autoclaving, which was replaced in each flask. Reactions were run at a total mass of 50 g (that is, sum of substrate, water, enzyme, media and yeast input) and flasks were capped with stoppers and gas traps. The reaction medium contained 0.05 M citrate buffer, 10 g/l yeast extract, 20 g/l peptone as well as the appropriate dose of enzyme, and were inoculated with *Saccharomyces cerevisiae *strain D5A to achieve an initial optical density (measured at 600 nm) of 0.5, which corresponds to a cell mass concentration of approximately 1.2 g dry cell mass/l. Initial pH was 5.0 to 5.2 and was not controlled thereafter. Reaction flasks were incubated for 7 days at a temperature of 38°C and shaking rate of 130 rpm in a rotary shaker (Innova 4080 shaker, New Brunswick Scientific, Edison, NJ, USA) with the exception of brief periods at 3, 5 and 7 days when the flasks were removed from the shaker for sampling. A US National Institute of Standards and Technology (NIST)-certified thermometer was used to verify incubator temperature. The SSF experiments were performed over four experimental blocks, with duplicate control flasks included in each block to verify block-to-block performance reproducibility. All tested conditions were run in duplicate or triplicate.

For SSF experiments, a simplified method was used to estimate cellulose conversion to glucose. The yield of glucose from cellulose was back calculated from ethanol production using Equation 1:

(1) $$\xi =\frac{\left(E_{f}-E_{0}\right)∕0.51}{f_{IS,i}\times f_{G}\times 1.111}\times 100\%$$

where *ξ *= cellulose conversion to glucose (% theoretical), *E_f _*= final ethanol concentration (g/l), *E_0 _*= initial ethanol concentration (g/l), *f*_IS, i _= initial insoluble solids (washed PCS) concentration, dry basis (g/l), *f *_G _= cellulose fraction in the input insoluble solids (g/g), 0.51 = maximum theoretical mass yield of ethanol from glucose (g/g) and 1.111 = mass yield of glucose from cellulose (water of hydration) (g/g).

The numerator approximates the amount of glucose produced by enzymatic hydrolysis and the denominator represents the amount of potential glucose input to the system as cellulose. This equation assumes that liquid density and liquid volume remain constant over the course of the experiment and that ethanol is produced only from cellulose-derived glucose and at 100% of theoretical yield. These simplifying assumptions are strictly not valid at higher levels of insoluble solids as discussed by others [27-29]. Nonetheless, Equation 1 was used as an efficient if less rigorous method to assess relative enzyme performance in the SSF hydrolysis mode.

### Standalone enzymatic hydrolysis saccharification

Standalone saccharification experiments tested enzymatic hydrolysis performance under selected conditions using both PCS WS and washed PCS solids. Reactions of 100 g were carried out in 250 ml capped Schott media bottles, and selected conditions were also run at 500 g reaction mass in 1 l capped Schott media bottles to provide enough residual solids to permit compositional analysis to be performed on final (day 7) samples. The bottles were autoclaved empty, but were not reautoclaved after solids and sterile water were added. The pH-adjusted PCS WS or washed PCS solids were manually introduced into the bottles using a small funnel to reach the target total solids concentration of 20% w/w (WS substrate) or 10.4% w/w (washed PCS substrate) accounting for sterile water and enzyme additions. These solids levels correspond to cellulose loading of 6.2% w/w for both systems. Initial flask pH was 5.0 to 5.2 and pH was not controlled thereafter.

Enzymatic saccharification reactions were started by adding enzyme to achieve the target enzyme dosage and then placing the fully loaded and capped bottles in a shaking incubator operating at 150 rpm and 50°C. The experiments were run for 7 days, with time course samples taken at 0, 3, 5 and 7 days for 100 g scale experiments, whereas the 500 g scale experiments were only sampled at 0 and 7 days. Multiple blank or 'dummy' bottles were incorporated into each experimental block to determine initial (time 0) total solids and insoluble solids levels as well as initial total sugar concentrations. A NIST-certified thermometer was used to verify shaker incubator temperature. The 100 g scale experiments were performed over five experimental blocks, with either duplicate or triplicate control flasks included in each block to verify block-to-block reproducibility. The 500 g scale experiments were carried out in two blocks in which each selected condition was run in duplicate, with complete compositional analysis of both solid and liquid fractions performed at the start (day 0 = dummy flasks) and at the end (day 7 = 168 h) of each experiment.

Enzyme performance (cellulose conversion yield) during enzymatic saccharification was quantified by the production of monomeric glucose as calculated by Equations 2 and 3:

(2) $$\xi =\frac{\Delta G}{f_{IS,i}\times f_{G}\times 1.111}\times 100\%$$

(3) $$\Delta G=\frac{g_{f}\cdot \left(1-f_{is,f}\right)}{\rho_{f}}-\frac{g_{i}\cdot \left(1-f_{is,i}\right)}{\rho_{i}}$$

where *ξ *= cellulose conversion to glucose (% theoretical), Δ*G *= change in glucose concentration (g/kg slurry), *g_f _*= final glucose concentration (g/l), *g_i _*= initial glucose concentration (g/l), *f*_is, i _= initial fraction insoluble solids (g/g), *f*_is, f _= final fraction insoluble solids (g/g), *f*_G _= cellulose fraction in the input insoluble solids (g/g), *ρ_i _*= initial liquid density (g/ml), *ρ_f _*= final liquid density (g/ml) and 1.111 = mass yield of glucose from cellulose (water of hydration) (g/g).

These equations are based on a mass balance across the system and account for liquid density and liquid volume changing over the course of the experiment. The numerator of Equation 2 represents the amount of glucose produced by enzymatic hydrolysis and the denominator represents the amount of potential glucose input to the system as cellulose.

### Soluble components analysis for SSF

Sugar concentrations were measured by high performance liquid chromatography (HPLC) using a Shodex SP0810 carbohydrate column (Shawa Denko KK, Kawasaki, Japan) and de-ashing guard cartridges (BioRad Laboratories, Hercules, CA, USA) following NREL standard laboratory analytical protocols [35,36]. Ethanol, acetic acid, hydroxymethylfurfural (HMF) and furfural were measured by HPLC using a Phenomenex Rezex RFQ Fast Fruit H+ organic acid column and Cation H+ guard cartridge (BioRad Laboratories) also following NREL standard laboratory analytical procedures [35]. Mixed component certified standards were periodically run between experimental samples to confirm HPLC calibration accuracy.

### Liquid density

Density of the liquid fraction was measured on supernatants recovered after sample centrifugation, following filtration through a 0.2 micron syringe filter, using an Anton-Parr model DMA-500 density meter (Ashland, VA, USA).

### Insoluble solids fraction

Triplicate measurements of insoluble solids fraction were performed, using standard NREL analytical procedures, on the initial dummy flask sample and duplicate measurements at the final sample point [35]. The slurry solids were dewatered by centrifugation and then the wet solid fraction was washed with deionized water until the concentration of glucose in the wash liquor fell to less than 0.05 g/l. Insoluble solids were then determined by drying washed samples at 45°C in a vacuum oven (0.6 bar) until they reached constant weight.

### Soluble components analysis for EH

Initial and final samples collected during the EH tests were analyzed by HPLC using NREL standard analytical procedures to determine concentrations of soluble sugars (monosaccharides and cellobiose) and total sugars (after secondary acid hydrolysis to measure combined monosaccharides and oligosaccharides), as well as carbohydrate degradation products and sugar alcohols [35,36]. Analysis was performed on sample supernatants, obtained by centrifugation, following filtration through 0.2 micron syringe filters.

### Solids compositional analysis

Compositional analysis of the PCS substrate was performed as described using standard NREL analytical procedures [35,36]. Briefly, the insoluble portion of a slurry sample was recovered, washed, dried and then subjected to a two-stage sulfuric acid hydrolysis process to solubilize the hemicellulose and cellulose sugars. The carbohydrates and acetyl content of the resulting analytical hydrolysate liquor were determined by HPLC as described previously. The complete step-by-step procedure is described in the reference [35] and is also available at NREL's website http://www.nrel.gov/biomass/analytical_procedures.html.

## Competing interests

The authors declare that they have no competing interests.

## Authors' contributions

JDM conceived and supervised the study, meta-analyzed the data, and drafted the paper. EWJ codesigned and oversaw execution of the EH and SSF experiments, performed EH experiments and contributed to writing and reviewing the paper. AM carried out enzyme protein and activity assays, and contributed to writing the Methods. MZ carried out the SSF experiments, assisted with EH experiment preparation, and contributed to the writing of the Methods. All authors read and approved the final manuscript.
